# Neural dynamics of the speed-accuracy trade-off

**DOI:** 10.1186/1471-2202-15-S1-P203

**Published:** 2014-07-21

**Authors:** Dominic Standage, Da-Hui Wang, Gunnar Blohm

**Affiliations:** 1 Department of Biomedical and Molecular Sciences, Queen’s University, Kingston, Canada; 2 Department of Systems Science, Beijing Normal University, Beijing, China

## 

**Figure 1:**
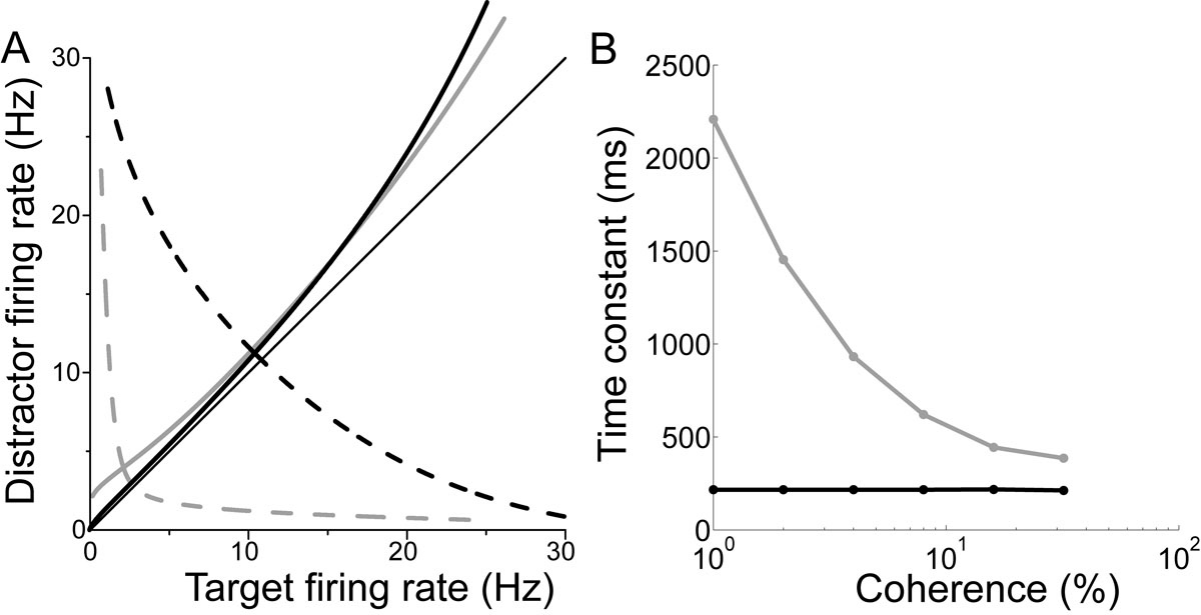
**A.** Stable (solid) and unstable (dashed) manifolds of the saddle point under speed (black) and accuracy (grey) conditions for one level of task difficulty (4% coherence). **B**. Time constant of the unstable manifold of the saddle for different task difficulties under speed and accuracy conditions. Coherence refers to the percentage of coherently moving random dots in a simulated decision task, where easier tasks have higher coherence.

In decision making experiments, faster decisions are accomplished at the expense of accuracy. Conversely, more accurate decisions are accomplished at the expense of time (see [1]). These data describe the speed-accuracy trade-off (SAT) and can be explained by the principles of bounded integration, where noisy evidence for the decision alternatives is integrated until one of the integrals reaches a bound. Higher bounds therefore support slower, more accurate decisions. These computations are widely believed to be implemented by feedback inhibition between neural populations selective for the alternatives. With the onset of evidence, the state of such a network is drawn toward a ‘saddle point’ (the intersection of the solid and dashed curves in Figure 1A) along a stable manifold (solid curves), from which it is repelled along an unstable manifold (dashed curves) toward an attractor corresponding to one of the alternatives (ends of the dashed curves). In the attractor state, the ‘winning’ population fires at a high rate and the other populations fire at low rates, consistent with electrophysiological data from decision tasks (see [2]). The dynamics in the vicinity of the saddle point are slow, supporting temporal integration. The SAT can therefore be controlled by mechanisms that modulate the dynamics near the saddle. Spatially non-selective excitation provides such a mechanism [3]. In simulations of a two-choice random dot motion task, we use this mechanism to control the SAT in a biophysically-based cortical model. A stronger non-selective signal leads to faster, less accurate decisions for two reasons: it shortens the time constant of the unstable manifold (Figure 1B) lowering decision time; and it re-positions the stable manifold, pushing it closer to the midline for rates below the saddle point. Thus, it shrinks the basin of attraction of the population receiving more evidence, so noise is more likely to drive the network toward the other (incorrect) attractor, lowering accuracy. The model accounts for recent electrophysiological recordings from putative integrator neurons showing a higher baseline rate, a higher rate of increase, and a higher peak under speed conditions (vice versa for accuracy) [4]. The model predicts that the firing rates of neurons selective for the alternatives will separate at higher rates under speed conditions, and that the difference between this rate and the baseline rate will be greater under speed conditions. Since the rates at the time of separation can be considered a ‘decision threshold’, the model conflicts with the hypothesis that a flexible bound is implemented by reducing the ‘threshold-baseline distance’ under speed conditions [1]. Our study suggests that a flexible bound is implemented by the rate at which decision dynamics unfold, not firing rates per se.
